# Role of Preoperative Chemoradiotherapy in Clinical Stage II/III Rectal Cancer Patients Undergoing Total Mesorectal Excision: A Retrospective Propensity Score Analysis

**DOI:** 10.3389/fonc.2020.609313

**Published:** 2021-01-18

**Authors:** Jii Bum Lee, Han Sang Kim, Ahrong Ham, Jee Suk Chang, Sang Jun Shin, Seung-Hoon Beom, Woong Sub Koom, Taeil Kim, Yoon Dae Han, Dai Hoon Han, Hyuk Hur, Byung Soh Min, Kang Young Lee, Nam Kyu Kim, Yu Rang Park, Joon Seok Lim, Joong Bae Ahn

**Affiliations:** ^1^Division of Medical Oncology, Department of Internal Medicine, Yonsei Cancer Center, Yonsei University College of Medicine, Seoul, South Korea; ^2^Division of Hematology-Oncology, Department of Internal Medicine, Ewha Womans University Medical Center, Ewha Womans University College of Medicine, Seoul, South Korea; ^3^Radiation Oncology, Yonsei Cancer Center, Yonsei University College of Medicine, Seoul, South Korea; ^4^Division of Gastroenterology, Department of Internal Medicine, Yonsei Cancer Center, Yonsei University College of Medicine, Seoul, South Korea; ^5^Department of Surgical Oncology, Yonsei Cancer Center, Yonsei University College of Medicine, Seoul, South Korea; ^6^Department of Biomedical Systems Informatics, Yonsei University College of Medicine, Seoul, South Korea; ^7^Department of Radiology, Yonsei Cancer Center, Yonsei University College of Medicine, Seoul, South Korea

**Keywords:** stage II/III, rectal cancer, total mesorectal excision, upfront surgery, chemoradiotherapy

## Abstract

**Background:**

Although the current standard preoperative chemoradiotherapy (PCRT) for stage II/III rectal cancer decreases the risk of local recurrence, it does not improve survival and increases the likelihood of preoperative overtreatment, especially in patients without circumferential resection margin (CRM) involvement.

**Methods:**

Stage II/III rectal cancer without CRM involvement and lateral lymph node metastasis was radiologically defined by preoperative magnetic resonance imaging (MRI). Patients who received PCRT followed by total mesorectal excision (TME) (PCRT group) and upfront surgery (US) with TME (US group) between 2010 and 2016 were analyzed. We derived cohorts of PCRT group versus US group using propensity-score matching for stage, age, and distance from the anal verge. Three-year relapse-free survival rate, disease-free survival (DFS), and overall survival (OS) were compared between the two groups.

**Results:**

A total of 202 patients were analyzed after propensity score matching. There were no differences in baseline characteristics. The median follow-up duration was 62 months (interquartile range, 46–87). There was no difference in the 3-year disease-free survival rate between the PCRT and US groups (83 vs. 88%, respectively; p=0.326). Likewise, there was no significant difference in the 3-year OS (89 vs. 91%, respectively; p=0.466). The 3-year locoregional recurrence rates (3 vs. 2% with US, p=0.667) and distant metastasis rates (16 vs. 11%, p=0.428) were not significantly different between the two groups. Time to completion of curative treatment was significantly shorter in the US group (132 days) than in the PCRT group (225 days) (p<0.001).

**Conclusion:**

Using MRI-guided selection for better risk stratification, US without neoadjuvant therapy can be considered in early stage patients with good prognosis. PCRT may not be required for all stage II/III rectal cancer patients, especially for the MRI-proven intermediate-risk group (cT1-2/N1, cT3N0) without CRM involvement and lateral lymph node metastasis. Further prospective studies are warranted.

## Introduction

Colorectal cancer (CRC) is the third most commonly diagnosed cancer worldwide and the second cause of cancer related death (1). The incidence of CRC is rising globally due to increase in western diet (2). Currently, the standard treatment for stage II/III rectal cancer is preoperative chemoradiotherapy (PCRT) followed by surgery and adjuvant chemotherapy (3). PCRT is effective in reducing local recurrence and down staging locally advanced rectal cancer (4, 5). However, it is associated with complications such as bowel (6), anorectal (7), and sexual dysfunctions (8, 9) and delay from surgical recovery (10). Although many patients benefit from local control of PCRT, there is still debate over whether it improves overall survival (11, 12). About one-third of patients relapse with metastasis despite treatment with PRCT and surgery (13).

Currently, National Comprehensive Cancer Network (NCCN) guidelines recommend preoperative chemoradiotherapy for tumors that are 1) T3, any N with clear CRM and 2) T1-2, N1-2  (14). In recent years, the incorporation of magnetic resonance imaging (MRI) in the preoperative setting has helped in better identification of tumor characteristics that dictate treatment strategies (15). Identification of features such as negative CRM, substaging T3, extramural venous invasion and nodal status in rectal cancer has helped treatment decisions in rectal cancer. In addition, the limitations of PRCT have led investigators to design trials that may omit PRCT in treatment of rectal cancer. Both the MERCURY (16) and OCUM (17) studies showed that rectal MRI could be used as an indicator to predict prognosis prior to surgery, thereby adjusting treatment according to patient’s prognosis. The QuickSilver study further addressed this issue by selecting MRI-predicted good prognosis subjects that resulted in a low rate of positive circumferential resection margin (CRM) (18). This study suggested the possibility of omitting PCRT in subjects with a negative CRM and no lateral lymph node metastasis in stage II/III rectal cancer.

The identification of subjects who do not need PCRT in an important issue in stage II/III rectal cancer. If adequate local control can be achieved by surgery alone, omitting PRCT may save patients from unnecessary chemotherapy and radiotherapy, thereby decreasing the time from diagnosis to surgery and without the associated complications from PRCT. Whether upfront surgical resection without PCRT is feasible for the above-defined subset of patients warrants further evidence.

In this study, we retrospectively selected MRI-proven intermediate-risk group (cT1-2/N1, cT3N0) patients without CRM involvement or lateral lymph node metastasis. The patients either received PRCT followed by TME (PCRT group) or underwent upfront radical surgery (US group). After selecting patients using propensity score analysis, we assessed the clinicopathological characteristics, disease free survival (DFS), overall survival (OS), and cumulative incidence of local and distant recurrence between the two groups. The primary objective of our study was to evaluate the 3-year DFS and OS between these two groups. Our hypothesis was that DFS and OS of upfront surgical resection are non-inferior to those of PCRT.

## Materials and Methods

### Study Design and Population

This was a retrospective study of stage II and III rectal cancer patients who received either PCRT followed by total mesorectal excision (TME) or underwent upfront radical surgery in Yonsei Cancer Center. Standardized rectal cancer MRI protocol was used for assessment of patients initially diagnosed with rectal cancer (18). Key inclusion criteria included (1) histologically confirmed stage II/III rectal adenocarcinoma with distance from anal verge ≤ 10 cm, (2) MRI predicted circumferential resection margin (CRM) greater than 1mm away from primary tumor, (3) extramural depth of invasion (EMD) ≤5mm, (4) absent extramural venous invasion (EMVI), (5) without pelvic lymph node involvement, (6) without distant metastasis, (7) surgery with TME, and (6) 3-year surveillance period after surgical resection. Patients with T1 and T2 tumors with N0 status, and patients requiring intersphicteric resection or abdominal perineal resection (APR) were excluded from the study.

The clinicopathologic variables such as age, gender, tumor grade, preoperative and postoperative MRI, clinical staging, types of surgery, pathologic staging, toxicity profiles of radiotherapy, and patterns of recurrence were collected. Staging was determined using the 8^th^ edition of the American Joint Committee on Cancer guideline of tumor, node, and metastasis (TNM) classification (19). CRM negative was defined as distance to the mesorectal fascia greater than 1 mm from the primary tumor (18).

Propensity score method was used to balance covariates and minimize bias (20, 21). Covariates included 1) clinical T stage 2) clinical N stage 3) primary location from the anal verge and 4) carcinoembryonic antigen (CEA) levels. Patients with missing data were excluded from the analysis.

This study was conducted in accordance with the Declaration of Helsinki. All patients provided written informed consent. The study was approved by institution review board of Yonsei Cancer Center (IRB 4-2020-0209).

### Treatment and Assessment

Baseline and follow up MRI were acquired on a 3.0-T system (MAGNETOM TrioTim; Siemens, Erlangen, Germany), and T2-weighted images of sagittal, axial, and oblique view were assessed (22). Further details of MRI techniques are discussed in previous protocols (23).

Patients diagnosed with stage II/III rectal cancer were either treated with upfront radical surgery only or PCRT followed by surgery. The 3-dimensional conformal radiotherapy (3D-CRT) was given at 45 Gy in 25 fractions over the course of 5 weeks, preoperatively for the PCRT group, and postoperatively for US group. During treatment, the following chemotherapy agents were given: intravenous 5-fluorouracil (425 mg/m^2^) and leucovorin (20mg/m^2^) were given as bolus on weeks 1 and 5, or capecitabine 1,650mg twice a day throughout radiation treatment. Interim assessment after PCRT was assessed with MRI for adaptation of surgical plan, and surgery was planned 6–8 weeks after completion of PCRT. In the US group, MRI was done at 4–6 weeks after surgery.

For both the PCRT and US group, patients were treated with adjuvant chemotherapy regimen such as FOLFOX (bolus and infused fluorouracil with oxaliplatin) and CAPOX (capecitabine and oxaliplatin) over the course of 6 months. Patients treated with FOLFOX were given oxaliplatin 85mg/m^2^ for 2 h with leucovorin 350mg intravenously, followed by bolus of fluorouracil 400mg/m^2^ on day 1 and infusion of fluorouracil 2,400mg/m^2^ over 2 days. Treatment was repeated every 2 weeks with total of 12 cycles in 6 months. CAPOX was given with 1,000mg/m^2^ of capecitabine twice per day for the first 14 days, and intravenous oxaliplatin 85mg/m^2^ over 2 h on day 1. The cycle was repeated every 3 weeks with a total of 8 cycles over 6 months.

Six weeks after surgery, patients who were candidates for adjuvant chemotherapy and radiotherapy received treatment accordingly. All patients followed up with computed tomography (CT) scans and CEA levels every 3 months for the first 2 years, and every 6 months for the next 3 years thereafter. Follow up colonoscopy was done at 1 year, 3 years and 5 years after surgery. Local recurrence was defined as tumor relapse within pelvis and perineum. Distant metastasis was defined as tumor recurrence outside locoregional area.

### Outcomes

Primary endpoints were 3-year DFS and OS in patients of rectal cancer with stage II/III disease who received either PCRT or underwent upfront radical surgery. Secondary endpoint was recurrence rate between two groups.

### Statistical Analysis

We analyzed data using Statistical Package for the Social Sciences (SPSS) version 25 (IBM, Chicago, IL) and the statistical software R (https://www.r-project.org, v3.5.0). Propensity score matching was performed to control potential confounding bias (20). The matching was constructed using clinical T stage, clinical N stage, primary location from the anal verge and CEA levels using the “MatchIt” R package. The nearest neighbor method was used with a caliper of 0.20 (**Supplementary Figures 1A, B**). Further, we conducted a sensitivity analysis for the matching estimate using “rbounds” R package, suggested by Rosenbaum (23). Briefly, sensitivity analysis for matched data evaluated the magnitude of potential bias using the Wilcoxon signed rank test. When gamma Γ (log odds of differential assignment to treatment due to unobserved factors) = 1, it holds assuming there is no hidden bias due to an unobserved confounder (**Supplementary Figure 1C**).

The correlations between variables were analyzed using Fischer’s exact test for categorical variables and sample *t*-test for continuous variables. Kaplan Meier with log-rank test was used to analyze survival difference between the two groups. Disease-free-survival (DFS) was defined as the time interval between surgery and tumor recurrence or last follow-up. Overall survival (OS) was defined as the time interval between the surgery and death or last follow-up.

## Results

### Patient Characteristics

**Figure 1:**
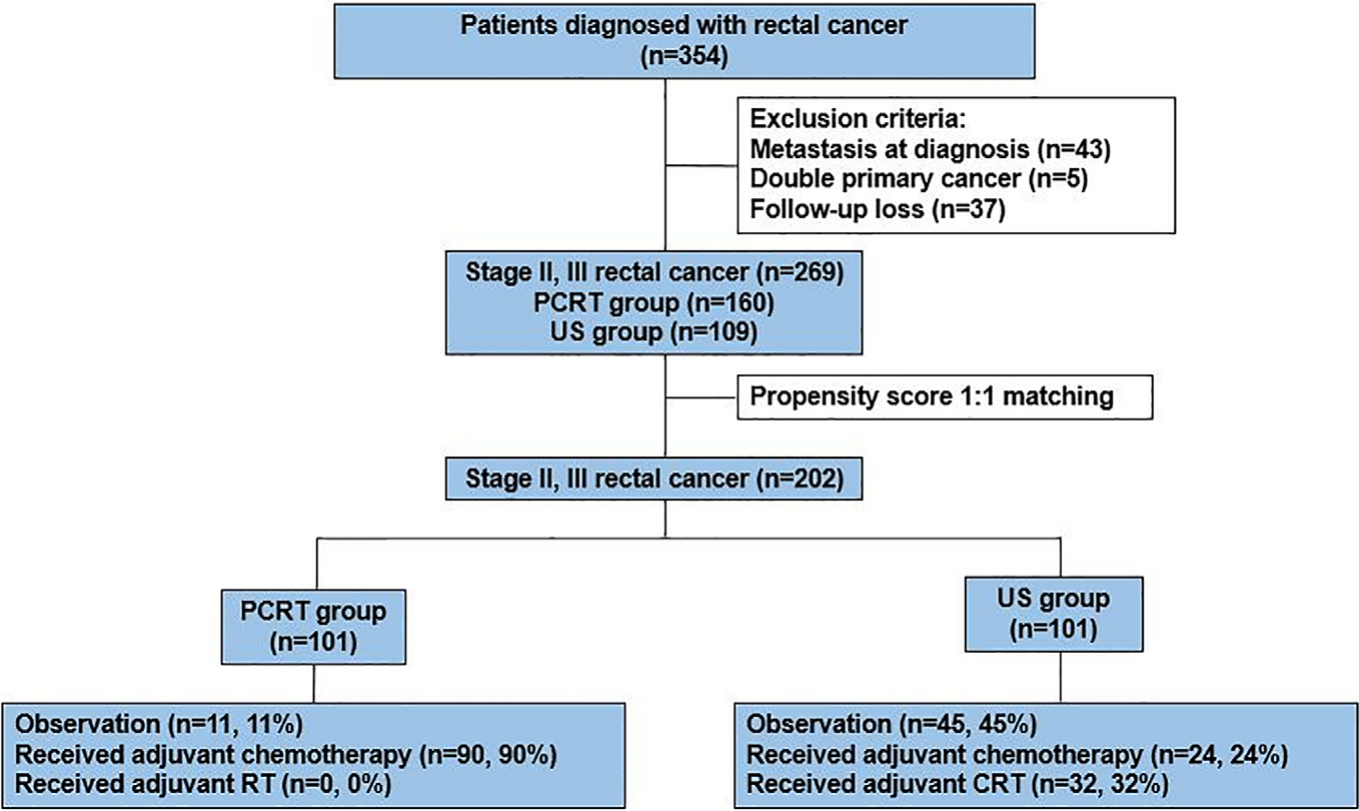
Study scheme. PCRT, Preoperative chemoradiotherapy; US, Upfront surgery.

Between January 2010 to June 2016, a total of 354 patients were diagnosed with rectal cancer in Yonsei Cancer Center. After excluding patients who had metastatic sites (n=43), double primary cancer (n=5), and incomplete data set due to follow up loss (n=37), data of 269 patients [PCRT, n=160 (59%); US, n=109(41%)] were collected. Since PRCT patients had relatively poorer prognostic factors such as high CEA and advanced clinical stage as compared to the US group, we used propensity score to adjust baseline characteristics between the two groups. After propensity score matching of 1:1 ratio, a total of 202 patients were selected for analysis with 101 patients from each group (**Figure 1**). All of the patients in the PCRT group completed planned cycles of pre-operative chemoradiotherapy without dose modifications.

### MRI-Based Tumor Characteristics and Histopathological Tumor Staging

Preoperative clinicopathological characteristics such as age, clinical stage, CEA level, and tumor location were well balanced between the two groups (**Table 1**). Most of the patients underwent lower anterior resection (LAR) [PCRT, n=96 (96%); US, n=96 (96%)]. Other surgical methods included ultralow anterior resection [PCRT, n=5(5%); US, n=3(3%)], transanal endoscopic operation (US, n=1) and total colectomy (US, n=1).

**Table 1:** Baseline patient characteristics.

Characteristics		PCRT Group	US Group	p value
No. (%)		n = 101	n = 101	
Age, years, Median (IQR)				
	< 70	55 (51–63)	57 (49–62)	0.87
	≥ 70	73 (70–76)	74 (72–78)	
Gender				
	Male	73 (73%)	65 (65%)	0.226
	Female	28 (28%)	36 (36%)	
CEA, ng/dl				
	< 5	64 (64%)	76 (76%)	0.067
	≥ 5	37 (37%)	25 (25%)	
Tumor location, from anal verge, cm				
	≤ 5	12 (12%)	12 (12%)	1
	> 5	89 (89%)	89 (89%)	
Tumor grade				
	WD	14 (14%)	9 (9%)	*0.743
	MD	83 (83%)	87 (87%)	
	PD	2 (2%)	3 (3%)	
	Unknown	2 (2%)	2 (2%)	
MRI findings				
	cT stage			
	cT1, 2	15 (15%)	23 (23%)	0.132
	cT3	86 (86%)	78 (78%)	
	cN stage			
	N0	28 (28%)	39 (39%)	0.182
	N+	73 (73%)	62 (62%)	
	pT category			
	pT0	23 (23%)	0 (0%)	0
	pTis	2 (2%)	1 (1%)	
	pT1	7 (7%)	8 (8%)	
	pT2	35 (35%)	36 (36%)	
	pT3	34 (34%)	56 (56%)	
	pN category			
	pN0	83 (83%)	62 (62%)	0.001
	pN1	14 (14%)	32 (32%)	
	pN2	4 (4%)	7 (7%)	
	pStage			
	Stage I, T1-T2, N0	58 (58%)	33 (33%)	0
	Stage II, T3-4, N0	25 (25%)	29 (29%)	
	Stage III, any T, N1-N2	18 (18%)	39 (39%)	
Type of surgery				
	LAR	96 (96%)	96 (96%)	*0.499
	ULAR	5 (5%)	3 (3%)	
	Other surgery§	0 (0%)	2 (2%)	
Recurrence				
	No	84 (84%)	89 (89%)	0.316
	Yes	17 (17%)	12 (12%)	
Pattern of recurrence				
	Local recurrence	3 (3%)	0 (0%)	*0.364
	Distant	13 (13%)	11 (11%)	
	Local + Distant	1 (1%)	1 (1%)	
Surgery (TME)				
	Complete	101 (100%)	101 (100%)	
	Incomplete	0 (0%)	0 (0%)	

^§^Patients who received with “Other surgery” include 1 transanal endoscopic operation, 1 total colectomy.

*Fisher’s exact test.

PCRT, preoperative chemoradiotherapy; US, upfront surgery; IQR, interquartile range; CEA, carcinoembryonic antigen; WD, well differentiated; MD, moderately differentiated; PD, poorly differentiated; MRI, magnetic resonance imaging; cT, clinical tumor; cN, clinical node; pT, pathologic tumor; pN, pathologic node; pStage, pathologic stage; LAR, lower anterior resection; ULAR, ultralow anterior resection; TME, total mesorectal excision.

In the PRCT group, there was significant down staging of postoperative pathologic stage (ypStage) after pre-operative chemoradiotherapy. A total of 23 patients (23%) in this group had a complete response (pT0) (p=0.000) and 83 patients (83%) with negative lymph nodes (p=0.001). Overall, PRCT group had higher proportion of stage I (n=58, 58%, p=0.000) and lower percentage of stage III (n=18, 18%) compared to the US group of stage I (n=33, 33%) and stage III (n=39, 39%), respectively.

Overall, there was no difference in recurrence at 3-years between the two groups [PRCT, n=17 (17%); US, n=12 (12%)] (p=0.316). Patterns of recurrence were also similar between the two groups, indicating that although PCRT group may have lower TNM stages, it had no effect on local [PCRT, n=3 (3%); US, n=0], distant [PCRT, n=13(13%); US, n=11(11%)] and combined local and distant recurrence [PCRT, n=1(1%); US, n=1(1%)] at 3 years (p=0.364). After TME, majority (n=83, 83%) of the PRCT patients did not receive treatment while 56% of US group required further treatment such as adjuvant chemoradiotherapy (CRT) (n=32, 32%) and adjuvant chemotherapy (n=24, 24%).

### Disease Free Survival, Overall Survival, and Local Recurrence Rate

**Figure 2:**
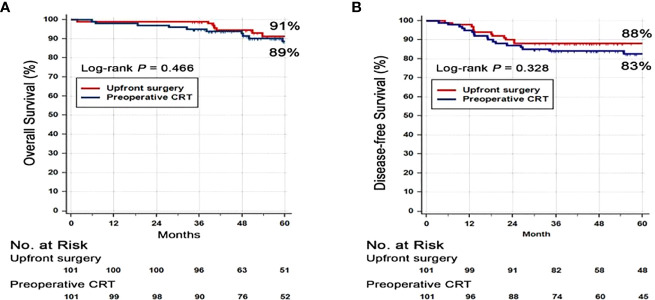
Kaplan-Meier survival curves for **(A)** disease free survival (DFS) and **(B)** overall survival (OS). Abbreviation: CRT, Chemoradiotherapy.

**Figure 3:**
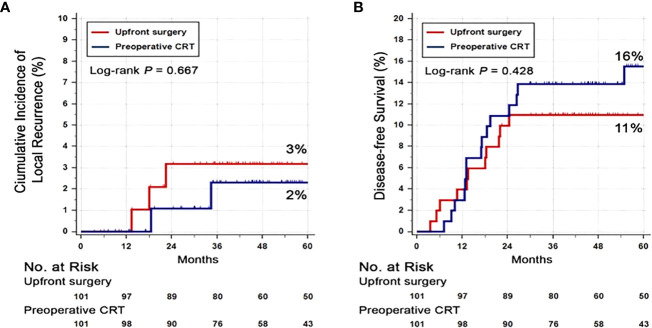
Cumulative incidence of **(A)** local recurrence and **(B)** distant recurrence. Abbreviation: CRT, Chemoradiotherapy.

At the data cut-off date of September 21, 2019, the median follow-up duration was 62 months (interquartile range, 46–87 months). Seventeen (17%) patients in the PRCT group and 8 patients (8%) in the US group had died. There was no difference in the 3-year DFS rate between PRCT group (83%) and US group (88%) (p=0.328) (**Figure 2**). No statistical difference in overall survival was seen between two groups; 3-year OS was 91 vs. 89% in US and PCRT group, respectively (p=0.466). Likewise, there was no difference in the rates of local and distant metastases. The rates of locoregional recurrence and distant metastasis at 3 years in PRCT and US groups were 3 vs. 2% (p=0.667) and 16 vs. 11% (p=0.428), respectively (**Figure 3**).

### Toxicity Profile

Among the PCRT group, 73 patients (73%) experienced adverse events of any grade (**Table 2**). The most common adverse events related to CRT were fatigue (n=42, 42%), diarrhea (n=42, 42%), and poor oral intake (n=41, 41%). In addition, fecal incontinence and tenesmus were seen in 18 patients (18%) and 9 patients (9%), respectively. The most common grade 3 adverse event was diarrhea (n=16, 16%). Of note, 1 patient (1%) had anastomotic leakage which required surgical intervention.

**Table 2:** Adverse events of PCRT and adjuvant CRT.

Adverse events		Any grade		Grade 3*	
		PCRT	Adjuvant CRT	PRCT	Adjuvant CRT
		n=101	n=32	n=101	n=32
No. (%)		73 (73%)	26 (79%)	19 (19%)	5 (15%)
*General*					
	Fatigue	42 (42%)	16 (48%)	1 (1%)	0 (0%)
	Nausea	1 (1%)	0 (0%)	0 (0%)	0 (0%)
	Poor oral intake	41 (41%)	0 (0%)	1 (1%)	0 (0%)
*Genitourinary Toxicity*					
	Cystitis	2 (2%)	0 (0%)	0 (0%)	0 (0%)
	Urinary incontinence	3 (2%)	0 (0%)	0 (0%)	0 (0%)
	Erectile dysfunction	4 (2%)	0 (0%)	0 (0%)	0 (0%)
*Gastrointestinal Toxicity*					
	Abdominal pain	3 (3%)	1 (3%)	0 (0%)	0 (0%)
	Anal pain	3 (3%)	0 (0%)	0 (0%)	0 (0%)
	Diarrhea	42 (42%)	11 (33%)	16 (16%)	5 (15%)
	Tenesmus	9 (9%)	1 (3%)	0 (0%)	0 (0%)
	Fecal incontinence	18 (18%)	5 (15%)	0 (0%)	0 (0%)
	Anastomotic leakage	1 (1%)	0 (0%)	1 (1%)	0 (0%)

PCRT, preoperative chemoradiotherapy; CRT, chemoradiotherapy; No., number.

*Grade ≥4 adverse events were not seen in both groups.

In the US group, 32 patients (32%) received adjuvant CRT, and 26 (79%) experienced adverse events of any grade including fatigue (n=16, 48%), diarrhea (n=11, 33%), and fecal incontinence (n=5, 15%). Grade 3 adverse event was only seen in diarrhea (n=5, 15%). Although the US group had higher incidence of adverse events of any grade, there was less grade 3 adverse events, and adverse events were manageable with supportive care. There were no adverse events of ≥ G4 or death due to CRT complications in both groups.

## Discussion

In our single center, retrospective study using propensity score analysis, we assessed the 3-year DFS and OS of preoperative chemotherapy followed by surgery versus upfront surgery in MRI proven, CRM negative stage II and III rectal cancer. Our study showed that although PCRT had significantly down-staged from the earlier postoperative pathologic stage, there was no difference in 3-year DFS, OS and cumulative incidence of local and distant recurrence between PRCT and US group. In addition, the retrospective analysis of adverse events due to CRT reflect that US groups had manageable toxicity of mostly grade 1–2. Compared to the PRCT group, the US group had less grade 3 adverse events which led to hospitalization, and surgical intervention for anastomotic leakage. Taken together, the oncologic outcomes of upfront-surgery group are comparable to those of PRCT group. With MRI directed patient stratification, it is possible for patients in intermediate-risk group (cT1-2/N1, cT3N0) without CRM involvement and lateral lymph node metastasis to omit PRCT, thereby avoiding overtreatment and reducing treatment duration.

Previously, there have been conflicting reports about the role of PCRT in locally advanced rectal cancer (5, 24–28). The Dutch trial showed that PRCT reduced the rate of local recurrence but had no impact on overall survival rate (26). Similarly, studies by German Rectal Cancer Study Group and Medical Research Council (MRC)-07 showed that although PRCT has a role in improving local control, there was no impact in overall survival (5, 24). Few studies have even pointed out that PCRT is unnecessary and may possibly be an overtreatment for some patients with stage II disease (27, 28).

Thereafter, several studies have proved that high-resolution rectal MRI has optimized the selection of CRM negative patient (16, 17). Good prognosis tumors treated with upfront surgery resulted in 2–5% of positive CRM patients (18). Even with upfront TME surgery alone, the 5-year local recurrences rates were as low as 4.4% (29). These findings are encouraging since patients without CRM involvement may selectively omit PCRT, thereby avoiding unnecessary complications from radiotherapy, save medical costs, and receive surgery without delay (30, 31).

Very few retrospective studies have addressed whether PCRT is essential in locally advanced rectal cancer. In a study comparing surgery alone and PRCT in recto-sigmoid junction cancer, PRCT was associated with 5% improvement of 5-year OS (32). However, that study included recto-sigmoid colon cancer. In contrast to the distal tumors that respond more favorably to PCRT, tumors located proximal from the anal verge respond less to PCRT (26). Therefore, the results with improved OS with PRCT in recto-sigmoid colon cancers must be interpreted with caution. In another study, early T3 rectal cancer patients who were either treated with surgery alone versus PRCT showed that 5-year local recurrence rate was 2% for both groups, and 5-year DFS were not statistically different (87% in surgery alone versus 88% in PRCT group) (33). Recently, a meta-analysis on total neoadjuvant therapy (TNT) addressed that TNT increases pathological down staging compared with surgery and adjuvant CRT (34). Despite higher rate of pathological complete response (pCR) by 39% in the TNT group, there was no difference in DFS and OS was noted between two groups.

Similarly, our study results proved that there was no difference 3-year OS between PRCT and US group. In addition, there was no difference in 3-year DFS and incidence of both local and distant recurrence between the two groups. Whether PCRT is a prerequisite in stage II/II rectal cancer, especially for MRI proven intermediate-risk group (cT1-2/N1, cT3N0) without CRM involvement and lateral lymph node metastasis, should be validated with prospective, randomized controlled trials in the future.

There are few limitations to our study. First, our study collected data retrospectively from a single center. Although we used propensity score matching to minimize cofounding covariates, variables such as physician’s choice for upfront surgery or PCRT, and patients’ treatment preferences may have inadvertently affected the allocation between two groups. Second, only the data from our institution was collected. Larger patient sampling from multi-centers in randomized controlled study (RCT) may provide additional information to clarify whether PCRT may be selectively avoided.

In conclusion, omitting PRCT and treatment with upfront surgery alone in CRM negative rectal cancer stage II/III patients may be considered as future treatment options. To further validate our retrospective results, a phase 2, randomized controlled trial of upfront surgery versus PRCT followed by surgery is currently ongoing (ClinicalTrials.gov, NCT02167321) (35).

## Data Availability Statement

The raw data supporting the conclusions of this article will be made available by the authors, without undue reservation.

## Ethics Statement

The studies involving human participants were reviewed and approved by institution review board of Yonsei Cancer 112 Center (IRB 4-2020-0209). The patients/participants provided their written informed consent to participate in this study.

## Author Contributions

JL, HK, AH, JC, SS, S-HB, WK, TK, YH, DH, HH, BSM, KL, YP, and NK participated in the collection of data. JSL reviewed radiologic findings, and HK and YP analysed the data. JL and HK drafted the manuscript. All the authors contributed to the article and approved the submitted version.

## Funding

This work was supported in part by the National Research Foundation of Korea (NRF) grant funded by the Korea government (MSIT) (No. 2019R1C1C1006709, No. 2018R1A5A2025079, and No. 2020M3F7A1094093), Basic Science Research Program through the National Research Foundation of Korea (NRF) funded by the Ministry of Education (2020R1F1A1066973), a grant of the Korea Health Technology Research and Development Project through the Korea Health Industry Development Institute (KHIDI), funded by the Ministry of Health & Welfare, Republic of Korea (No. KHIDIHI19C1015010020), "The Alchemist Project" through the Ministry of Trade, Industry and Energy (MOTIE, Korea) (20012443), Severance Hospital Research fund for Clinical excellence (SHRC) (C-2020-0032 and C-2020-0025).

## Conflict of Interest

The authors declare that the research was conducted in the absence of any commercial or financial relationships that could be construed as a potential conflict of interest.

## Supplementary Material

Supplementary Figure 1 Propensity score matching. **(A)** Distribution of propensity scores. **(B)** Histograms of propensity scores before and after matching. **(C)** Sensitivity analysis with matched data.
