# Supplementary figures and images for: Role of Preoperative Chemoradiotherapy in Clinical Stage II/III Rectal Cancer Patients Undergoing Total Mesorectal Excision: A Retrospective Propensity Score Analysis
Source: Front Oncol. 2021 Jan 18;10:609313. doi: 10.3389/fonc.2020.609313 (PMC7848147; doi:10.3389/fonc.2020.609313)

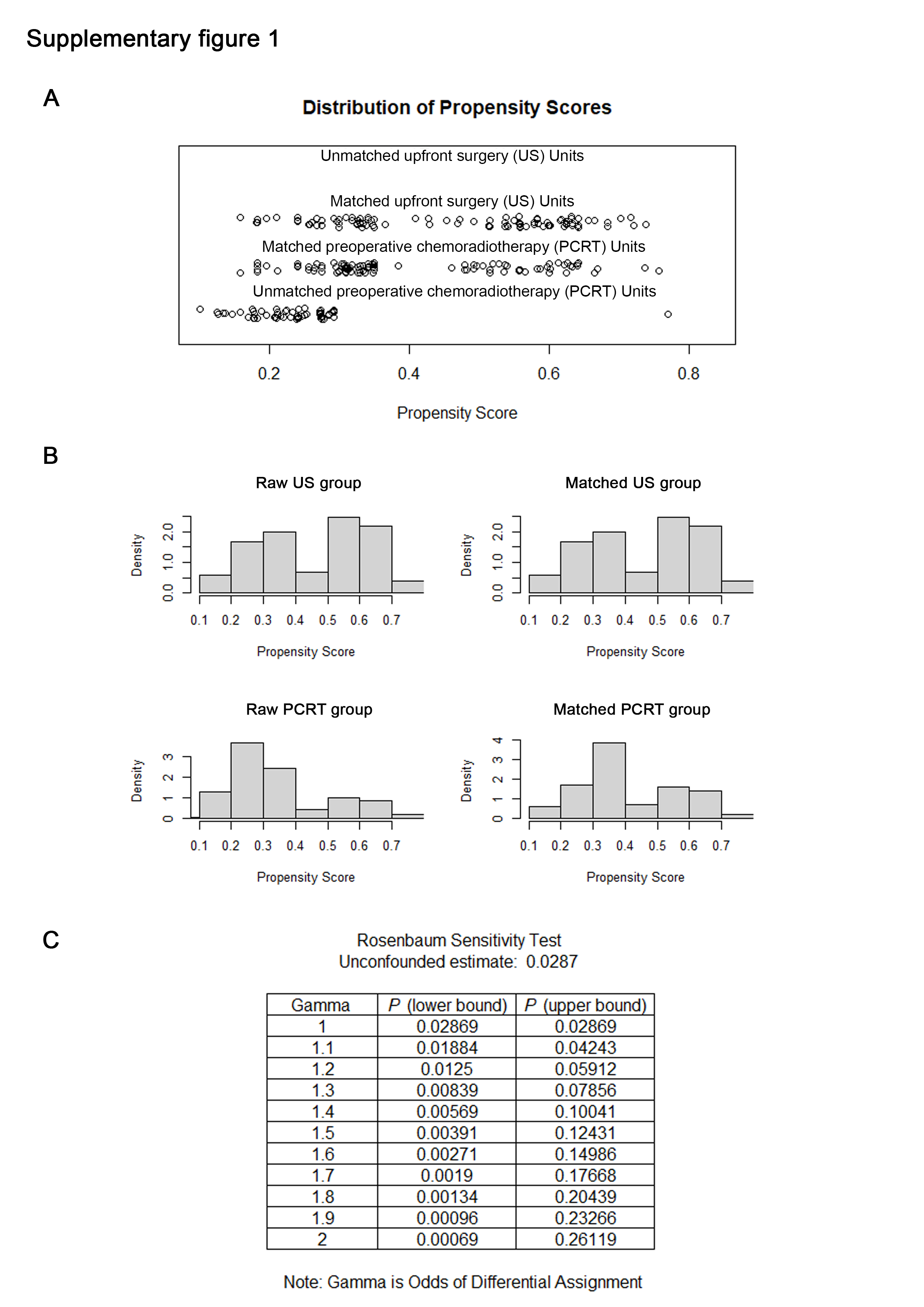

Supplement: Supplementary Figure 1 — Propensity score matching. (A) Distribution of propensity scores. (B) Histograms of propensity scores before and after matching. (C) Sensitivity analysis with matched data. [file Image_1.tif]
